# Bowel Preparation for Colonoscopy in Patients with Diabetes Mellitus—A Gap We Have to Bridge: A Review

**DOI:** 10.3390/jcm14103336

**Published:** 2025-05-11

**Authors:** Ivana Jukic, Jonatan Vukovic

**Affiliations:** 1 Department of Gastroenterology and Hepatology, University Hospital Split, Spinciceva 1, 21000 Split, Croatia; jonatan.vukovic@mefst.hr; 2 University Department of Health Studie, University of Split, 21000 Split, Croatia; 3 Department of Internal Medicine, School of Medicine, University of Split, 21000 Split, Croatia

**Keywords:** colonoscopy, diabetes mellitus, bowel preparation, guidelines, detection, adenoma, colon cancer, recommendations

## Abstract

Colonoscopy is an essential diagnostic and therapeutic tool in gastroenterology, significantly impacting colorectal cancer (CRC) detection and management. Effective bowel preparation is critical for optimal visualization, directly influencing colonoscopy accuracy and patient outcomes. However, diabetic patients frequently encounter challenges achieving adequate bowel preparation, primarily due to gastroparesis, autonomic neuropathy, altered colonic motility, fluid–electrolyte imbalances, and complexities related to antihyperglycemic medication adjustments. This review aims to evaluate the current literature on bowel preparation efficacy in diabetic patients undergoing colonoscopy, assess existing guidelines from leading gastroenterological societies, and highlight the necessity for detailed, diabetes-specific recommendations. We conducted a comprehensive PubMed search identifying 20 pertinent studies, including randomized controlled trials, meta-analyses, multicenter studies, cohort studies, and reviews. The findings consistently indicate diabetes as an independent predictor of inadequate bowel preparation. Furthermore, an evaluation of guidelines from the European Society of Gastrointestinal Endoscopy (ESGE), the US Multi-Society Task Force, and the Canadian Association of Gastroenterology revealed either absent or insufficiently detailed diabetes-specific recommendations. Given the rising global prevalence of diabetes and CRC, inadequate bowel preparation significantly impacts the quality of colonoscopy, adenoma detection rates, patient safety, and healthcare costs. This review underscores the urgent need for additional research focusing on tailored bowel preparation strategies for diabetic patients. Ultimately, the implementation of standardized, evidence-based protocols designed explicitly for this high-risk group is essential to enhance diagnostic efficacy, improve patient outcomes, and reduce CRC-related morbidity and mortality.

## 1. Introduction

Colonoscopy remains the cornerstone diagnostic and therapeutic intervention in gastroenterology, serving as a pivotal tool for the detection and resection of precancerous lesions, including adenomas, and for the diagnosis and management of various colonic pathologies. The efficacy of colonoscopy is inextricably linked to the adequacy of bowel preparation, which is essential for optimal mucosal visualization. High-quality bowel cleansing has been unequivocally associated with improved adenoma detection rates, a reduced likelihood of missed lesions, and a diminished need for repeat procedures [1]. Conversely, inadequate bowel preparation compromises diagnostic accuracy, prolongs procedural duration, elevates the risk of procedural complications, and exacerbates patient discomfort, collectively imposing a substantial economic and logistical burden on healthcare systems [2]. Despite its diagnostic utility, colonoscopy may miss a substantial proportion of colonic lesions, with studies reporting an adenoma miss rate of up to 26% [3]. Colonoscopy remains a cornerstone in the early detection and prevention of colorectal cancer (CRC). Its diagnostic yield is directly dependent on the adequacy of bowel preparation. Inadequate cleansing compromises mucosal visualization, reduces adenoma detection rates (ADR), and increases both procedure time and healthcare costs. Studies report that up to 25–30% of colonoscopies may be affected by suboptimal preparation [1,2]. High-risk groups, such as patients with type 2 diabetes mellitus (T2DM), are especially prone to inadequate bowel preparation due to motility disorders, autonomic neuropathy, and the influence of glucose-lowering medications. However, despite the growing global prevalence of both T2DM and CRC, clinical guidance remains limited and non-specific for diabetic patients [3,4].

Despite continuous refinements in bowel preparation regimens, achieving optimal colonic cleansing remains a formidable challenge, particularly in specific high-risk patient populations, among which individuals with diabetes mellitus (DM) constitute a distinct subgroup. Diabetic patients exhibit a disproportionately high risk of suboptimal bowel preparation due to a constellation of pathophysiological mechanisms, including delayed gastric emptying (gastroparesis), autonomic neuropathy, and altered colonic motility, all of which contribute to prolonged intestinal transit and impaired bowel cleansing. In comparison to non-diabetic individuals, patients with diabetes mellitus commonly exhibit delayed gastric emptying and impaired colonic transit, resulting in reduced bowel evacuation efficiency [4]. Moreover, systemic factors such as fluid and electrolyte imbalances, dietary restrictions, and the complexities of antihyperglycemic therapy modifications further compound the challenges associated with bowel preparation in this patient population.

According to the International Diabetes Federation (IDF), the IDF Diabetes Atlas (2021) reports that an estimated 537 million adults aged 20–79 years are currently living with diabetes, a figure projected to escalate to 643 million by 2030 and 783 million by 2045 [5]. Concurrently, colorectal cancer remains one of the most prevalent malignancies worldwide, ranking as the second most common cancer among women and the third most common among men [6].

Bearing in mind epidemiological data on the prevalence of diabetes and colorectal cancer in the world, and the importance of colonoscopy as a screening method, the goals of our article are as follows:

1. Search the literature on the topic of bowel preparation for colonoscopy in diabetics;
2. Determine whether the currently valid guidelines of the most important gastroenterology associations contain specific instructions on bowel preparation for colonoscopy;
3. Determine if there are possibly insufficiently detailed recommendations of the current guidelines and point out the importance and specifics of bowel preparation for colonoscopy in diabetics.

## 2. Materials and Methods

Regarding the fact that this article is a review article, we used the PubMed electronic database for the literature search.

To collect literature for our first research aim, we used the keywords “diabetes mellitus” AND “colonoscopy” AND “bowel preparation”, and 57 articles were found. For literature selection, we have used the PubMed filter option and selected only the following types of studies: clinical trial, controlled clinical trial, guideline, meta-analysis, multicenter study, observational study, practice guideline, books and documents, randomized controlled trial, review, and systematic review.

Specific inclusion criteria involved only human studies with adult and young study populations. Language other than English was not a limitation. The exclusion criteria were as follows: all other types of literature and articles listed among the search PubMed options, in vitro or animal studies, and articles without full-text availability. The full texts of manuscripts that appeared to be potentially relevant to our article were obtained and evaluated by both authors. The literature search covered all studies published until February 2025. Inclusion criteria included articles from January 2009 to February 2025. Language was not a restriction, and full-text availability was required. Studies that were in vitro, animal-based, or without accessible full text were excluded.

To collect the literature for our second study aim, we searched for current guidelines using the websites of important global gastroenterology associations and/or organizations.

## 3. Results

For our first aim, the article search identified twenty-two relevant full-text articles from the PubMed electronic database. Twenty of the twenty-two studies that met the full inclusion criteria for this article were retrieved and fully reviewed.

Summarizing all articles, five review articles, two meta-analyses, seven randomized controlled trials, two multicenter studies, and four cohort studies were included in this review [7,8,9,10,11,12,13,14,15,16,17,18,19,20,21,22,23,24,25,26]. The first study on this topic was published in 2009 by Ozturk NA et al., who concluded that optimal bowel cleansing is poorer in diabetics with autonomous neuropathy than in those without autonomous neuropathy and non-diabetic controls [26]. The same author published another clinically controlled study in 2010, where they investigated the safety and tolerability of sodium phosphate in diabetics, and concluded that colon preparation for colonoscopy is proportional to the duration of diabetes and the presence of late complications [25]. Hayes A et al. in 2011 found that colon preparation is better achieved with specialized protocols than with standardized ones [24]. Rotondano G et al. confirmed in 2015 that diabetes mellitus is an independent predictor of inadequate bowel cleansing, both at the level of the right and left colon, in the overall population [23]. Kim YH et al. in 2017 published the results of a randomized controlled trial, and the conclusion was that diabetic patients had a worse preparation quality and longer cecal intubation and total procedure time compared to non-diabetic patients [22].

Similarly, Alvarez-Gonzalez et al. (2016) showed that a diabetes-specific preparation protocol significantly improved bowel cleansing compared to a conventional one (inadequate preparation rates of 7% vs. 20%, *p* = 0.014) [20]. Zhang et al. (2024) conducted a meta-analysis confirming DM as an independent predictor of inadequate bowel preparation for colonoscopy (IBP), along with other risk factors like constipation and non-adherence to dietary regimens [8]. Additionally, the impact of GLP-1 (glucagon-like peptide-1) receptor agonists was explored by Abu-Freha et al. (2025), highlighting a significantly higher rate of IBP in diabetic patients treated with these agents. We would like to emphasize the importance of a recent publication from 2025 regarding the multicenter design of the study and the fact that it included almost 5000 participants with DM. This study not only confirmed that DM is an independent risk factor for IBP for colonoscopy but also identified GLP-1 RA as an additional contributing factor for IBP. These findings are highly relevant in light of the globally increasing prescription rate of GLP-1 RA [7].

In subsequent years, from 2019 to 2025, published articles have shown that diabetes mellitus is an independent risk factor/predictor of inadequate bowel preparation [7,8,12,14,16,17,18]. Bearing in mind that the results of this are provided by randomized clinical research and meta-analysis with a large number of patients, the value of such evidence should be considered significant. Among the twenty-two studies included, seven were randomized controlled trials, two were meta-analyses, four were cohort studies, two were multicenter studies, and five were narrative or systematic reviews, which consistently demonstrated a significantly higher rate of inadequate bowel preparation in patients with diabetes mellitus compared to non-diabetic controls.

See the results in Table 1.

For our second goal, we identified three articles/guidelines from the European Society of Gastrointestinal Endoscopy (ESGE) Guidelines; the US Multi-Society Task Force on Colorectal Cancer: American College of Gastroenterology, American Gastroenterological Association, and American Society for Gastrointestinal Endoscopy; and the Canadian Association of Gastroenterology.

For the results, please see Table 2.

## 4. Discussion

Globally, there is an alarming rise in the incidence of diabetes mellitus and colorectal cancer, marking both conditions as critical public health issues [5,6,30]. Diabetes mellitus, particularly type 2, is escalating in prevalence primarily due to modern lifestyle factors such as poor dietary habits, obesity, reduced physical activity, and increasing lifespan [31]. Concurrently, colorectal cancer remains one of the leading causes of cancer-related morbidity and mortality worldwide, exhibiting steadily rising incidence rates [6,30]. Recent studies consistently document an increased incidence of colorectal cancer among patients with diabetes compared to the non-diabetic population [32]. Several biological mechanisms have been suggested to explain this correlation, including hyperinsulinemia, insulin resistance, chronic inflammation, oxidative stress, and significant alterations in gut microbiota [33]. These metabolic disturbances create an environment conducive to carcinogenesis, amplifying CRC risk among diabetic patients. Colonoscopy represents a cornerstone of colorectal cancer screening and prevention. However, its efficacy is highly dependent on optimal bowel preparation. Poor bowel preparation significantly compromises the diagnostic accuracy of colonoscopies, prolongs procedure durations, increases healthcare costs, and necessitates repeat procedures [2]. Diabetic patients particularly struggle with adequate bowel preparation, primarily due to complications like gastrointestinal dysmotility, gastroparesis, and altered colonic transit times [4]. The adequacy of bowel preparation is often evaluated using the Boston Bowel Preparation Scale (BBPS). BBPS is a standardized, validated scoring system designed to assess bowel cleanliness in three colonic segments: the right colon, transverse colon, and left colon. Each segment is rated from 0 (inadequate, mucosa not visible) to 3 (excellent, mucosa clearly visible), resulting in a total score ranging from 0 to 9. Scores equal to or greater than 6, with at least 2 points per segment, indicate sufficient preparation quality [4]. Given the substantial diabetic population and their increased colorectal cancer risk, optimizing colonoscopy efficacy through improved bowel preparation is crucial. Surprisingly, the current literature and guidelines specific to bowel preparation in diabetic patients are sparse. A comprehensive search of the medical databases yielded only 20 studies addressing the specific challenge of bowel preparation among diabetic patients undergoing colonoscopy [7,8,9,10,11,12,13,14,15,16,17,18,19,20,21,22,23,24,25,26]. This limited volume of research is particularly concerning given the high global prevalence of both diabetes and colorectal cancer, highlighting a significant gap in clinical evidence. In light of this evidence gap, we advocate strongly for further research dedicated to the specific needs and physiological challenges of diabetic patients in bowel preparation. Enhanced research outcomes could provide critical insights that would inform and necessitate a revision of existing clinical guidelines. Specifically, targeted guidelines for diabetic patients would support improved clinical outcomes, patient safety, and cost-effectiveness. Although Canadian researchers previously addressed this concern and published recommendations aiming to improve bowel preparation among diabetic patients, these recommendations have unfortunately not been integrated into daily clinical practice nor formally incorporated into broader clinical guidelines [34]. This underscores the need for an active implementation strategy, ensuring recommendations transition effectively from research findings into routine practice. Ultimately, the revision of current bowel preparation guidelines to explicitly accommodate diabetic patients is imperative. The implementation of evidence-based, diabetes-specific bowel preparation protocols is critical to enhancing the diagnostic accuracy and effectiveness of colonoscopies, thereby facilitating timely CRC detection, better clinical management, and improved outcomes for this high-risk patient group.

The following text addresses the key issue of this topic.

### 4.1. Pathophysiological Mechanisms Affecting Bowel Preparation in Diabetic Patients

The mechanisms contributing to inadequate bowel preparation in diabetic patients are multifactorial. Gastroparesis, a frequent complication of long-standing diabetes, leads to delayed gastric emptying and altered intestinal motility, which, in turn, disrupts the efficacy of bowel cleansing solutions [35]. Autonomic neuropathy, another common consequence of diabetes, further impairs gastrointestinal motility, reducing colonic peristalsis and contributing to inefficient evacuation of bowel contents. Additionally, colonic dysbiosis associated with diabetes, characterized by alterations in gut microbiota composition, may further impact bowel preparation efficacy by influencing intestinal transit and fluid absorption [36].

Beyond motility disturbances, metabolic factors play a crucial role. Diabetic patients often experience dehydration due to polyuria associated with hyperglycemia, which can exacerbate fluid shifts induced by bowel cleansing agents. Electrolyte imbalances, particularly sodium and potassium disturbances, may further hinder colonic motility and increase the risk of adverse events associated with bowel preparation. Moreover, the need for dietary modifications prior to colonoscopy can be particularly challenging for diabetic patients who must carefully manage blood glucose levels. The requirement for a low-residue diet followed by a clear liquid diet may lead to fluctuations in glycemic control, increasing the risk of hypoglycemia or hyperglycemia.

### 4.2. Impact of Antihyperglycemic Medications on Bowel Preparation

Diabetes pharmacotherapy presents another layer of complexity in bowel preparation. Many antihyperglycemic agents have implications for fluid balance, gastrointestinal motility, and metabolic stability, necessitating careful medication adjustments in the peri-colonoscopy period. The Canadian Association of Gastroenterology provides specific recommendations regarding the management of these medications:

- Metformin should be discontinued upon initiation of a clear liquid diet due to its association with lactic acidosis risk [37].
- GLP-1 receptor agonists, which delay gastric emptying, should be withheld if a once-weekly dose is scheduled within two days before colonoscopy. This class of medications may exacerbate delayed bowel transit and contribute to inadequate cleansing [38].
- DPP-4 (dipeptidyl peptidase 4) inhibitors should be omitted on the morning of the procedure, as they have minimal risk of hypoglycemia but may still interact with fasting metabolism [39].
- SGLT-2 (sodium/glucose cotransporter 2) inhibitors should be stopped three days before colonoscopy to reduce the risk of dehydration and euglycemic ketoacidosis [40].
- Insulin therapy should be carefully adjusted, with dose reductions or omissions as appropriate, to prevent hypoglycemia during fasting [34].

These recommendations underscore the necessity for individualized bowel preparation protocols in diabetic patients, as the interplay between medication use, glycemic control, and bowel cleansing agents poses a unique set of challenges.

### 4.3. Clinical Implications of Inadequate Bowel Preparation

Suboptimal bowel preparation in diabetic patients not only diminishes procedural efficacy but also increases the likelihood of requiring repeat colonoscopies, thereby exposing patients to additional procedural risks and healthcare costs. Studies have consistently demonstrated that diabetic patients are nearly twice as likely as their non-diabetic counterparts to exhibit inadequate bowel preparation, with reported prevalence rates of up to 25% [2].

A poorly prepared bowel significantly reduces the adenoma detection rate, a key quality metric in colonoscopy, potentially leading to missed precancerous lesions. Given the increased risk of colorectal neoplasia in individuals with type 2 diabetes, failure to achieve adequate bowel preparation may delay the detection of malignancies, ultimately impacting colorectal cancer outcomes [41]. Furthermore, prolonged procedural times associated with inadequate preparation increase patient discomfort, elevate sedation requirements, and contribute to higher complication rates, including perforation and post-procedural bleeding.

### 4.4. The Burden of Diabetes and Colorectal Cancer

The importance of optimizing bowel preparation among diabetic patients is underscored by current global epidemiological patterns. According to the International Diabetes Federation (IDF) Diabetes Atlas (2021), approximately 537 million adults aged 20–79 currently have diabetes. This number is projected to rise significantly to 643 million by 2030 and further to 783 million by 2045 [5]. At the same time, colorectal cancer (CRC) continues to be among the most widespread cancers globally, ranking second in incidence among women and third among men. CRC represents about 10% of all new cancer cases and cancer-related deaths each year [6].

Notably, type 2 diabetes has been identified as a risk factor for colorectal adenomas, the benign neoplastic precursors to CRC, predominantly in populations with White/European ancestry [41]. The intersection of diabetes and colorectal cancer risk highlights the critical need for ensuring high-quality colonoscopy preparation in diabetic patients, as inadequate visualization may result in missed lesions, thereby exacerbating cancer-related morbidity and mortality. Patients with colorectal cancer (CRC) and concurrent type 2 diabetes mellitus (T2DM) experience a higher economic burden compared to non-diabetic patients. Notably, individuals with both T2DM and Stage II CRC face a significantly greater economic burden, while surgical patients exhibit a substantially higher disease burden than those managed non-surgically. Efforts should focus on both primary and secondary prevention strategies to mitigate the financial burden associated with colorectal cancer [42].

### 4.5. Chronic Low Grade Inflammation, Oxidative Stress, and Gut Microbiota Alterations in Patients with Type 2 Diabetes Mellitus

Emerging evidence has increasingly clarified the complex interplay between chronic inflammation, oxidative stress, and gut microbiota alterations in patients with type 2 diabetes mellitus (T2DM), all of which may have implications for bowel preparation efficacy. Diabetes-associated metabolic disturbances promote a pro-inflammatory state and oxidative imbalance, which can impair intestinal epithelial function and motility [43]. Furthermore, oxidative stress contributes to insulin resistance and mucosal dysfunction, exacerbating colonic dysmotility and potentially interfering with the effectiveness of standard bowel cleansing regimens. Gut microbiota composition in T2DM patients is frequently altered, characterized by a reduction in short-chain fatty acid-producing bacteria and an increase in pro-inflammatory species, which further perpetuate dysbiosis and intestinal transit delays. These factors collectively reduce the efficiency of bowel evacuation and may impair mucosal visualization during colonoscopy.

Given these mechanisms, personalized preparation strategies may be warranted, particularly for individuals with long-standing or poorly controlled diabetes. In line with this, antioxidant-based therapeutic approaches have been proposed as adjuncts in gastrointestinal management due to their potential to restore redox balance and mitigate oxidative damage in the GI tract [44]. Although still experimental, such approaches could eventually be integrated into bowel preparation regimens for high-risk populations. Ultimately, understanding the pathophysiological underpinnings of diabetes-related bowel dysfunction can inform more effective, targeted strategies for improving colonoscopy outcomes.

When optimizing bowel preparation for patients with type 2 diabetes mellitus, several clinically relevant considerations must be addressed. Evidence suggests that scheduling colonoscopies later in the morning improves adherence to split-dose regimens and reduces the risk of hypoglycemia during prolonged fasting. Diabetic patients frequently present with delayed gastric emptying, impaired colonic transit, and autonomic neuropathy, all of which impair bowel cleansing effectiveness. To counteract these physiological barriers, modified strategies such as extended preparation windows, split dosing, and the use of adjunctive agents like prokinetics may be beneficial. Patient education also plays a pivotal role, with studies showing that structured counseling and visual instructions significantly improve preparation outcomes and compliance. Moreover, particular attention must be paid to antihyperglycemic medications; for example, SGLT2 inhibitors and GLP-1 receptor agonists should be temporarily withheld, and insulin regimens should be adjusted to prevent metabolic complications. In patients with additional risk factors, such as gastroparesis or renal impairment, individualized protocols, including inpatient monitoring or alternative bowel cleansing agents, may be warranted. These considerations highlight the need for comprehensive, diabetes-specific bowel preparation guidelines that account for the complex pathophysiology and treatment regimens typical of this population.

### 4.6. The Need for Standardized and Evidence-Based Guidelines

A growing body of research highlights the inconsistencies in current bowel preparation guidelines for diabetic patients. Many of the available protocols are generalized and do not address the heterogeneity in diabetes management, comorbidities, and treatment strategies. The physiological challenges posed by diabetes necessitate a more structured and targeted approach to bowel cleansing. Furthermore, newer bowel preparation regimens, including split-dose and low-volume polyethylene glycol-based solutions, need further evaluation in diabetic populations to determine their efficacy and tolerability.

Our findings confirm the consistent association between diabetes mellitus and suboptimal bowel preparation for colonoscopy, as established in both prospective studies and meta-analyses. While this association is known, the clinical implication, namely the lack of comprehensive, tailored preparation protocols for diabetic patients, remains critically under-addressed. Despite robust evidence, leading guidelines such as those from the ESGE, the US Multi-Society Task Force, and the Canadian Association of Gastroenterology either omit or only briefly mention diabetic-specific recommendations. The absence of detailed, evidence-based guidelines limits the ability of clinicians to optimize colonoscopy outcomes for this growing high-risk population.

We also identified a key gap in how antihyperglycemic medications are managed in the peri-colonoscopy period. Drugs like GLP-1RAs and SGLT2 inhibitors have substantial implications for bowel motility and hydration, yet most protocols fail to address them. This review calls for urgent guideline revisions that incorporate specific pharmacologic, dietary, and scheduling considerations for diabetic patients.

Finally, the review emphasizes the need for structured patient education programs to improve adherence and preparation quality. Targeted communication strategies and diabetes-specific instructions could mitigate the risk of IBP and reduce repeat procedures, ultimately improving detection rates and reducing healthcare costs.

Future studies should also explore the role of patient education in improving adherence to bowel preparation instructions. The misinterpretation of dietary and medication guidelines remains a significant factor contributing to inadequate cleansing. Structured educational interventions, including pre-procedural counseling, written instructions, and digital resources, may improve compliance and preparation quality in diabetic patients.

## 5. Summary

A dedicated, prospective, multicenter clinical investigation is critically needed to systematically evaluate the comparative efficacy, tolerability, and safety profiles of diverse bowel preparation regimens, specifically in diabetic patients undergoing colonoscopy. Given the complex pathophysiological mechanisms unique to diabetic populations, including gastroparesis, autonomic neuropathy, altered gastrointestinal motility, fluid–electrolyte imbalances, and medication-related challenges, research efforts must focus on refining existing bowel cleansing strategies and exploring innovative preparations that effectively address these specific issues. Future studies should rigorously examine the effectiveness of split-dose and low-volume polyethylene glycol-based preparations, alongside novel osmotic and stimulant laxatives specifically adapted for diabetic patients with compromised gastrointestinal function. In parallel, comprehensive evaluations should be undertaken to assess the impact of structured patient education interventions and adherence enhancement strategies, including interactive multimedia educational tools, patient counseling, and personalized medication adjustment protocols. These measures have significant potential to improve patient compliance and, consequently, bowel cleansing outcomes. Moreover, investigations into emerging bowel preparation solutions designed explicitly for diabetic patients with impaired gastrointestinal motility should be prioritized. Innovations such as prokinetic adjunct therapies, novel formulations improving intestinal transit, and patient-specific dosing schedules should be explored and rigorously validated through randomized controlled trials. These tailored bowel preparation protocols could notably enhance the quality of mucosal visualization, adenoma detection rates, and overall diagnostic accuracy. The urgent development and formal implementation of comprehensive, evidence-based bowel preparation guidelines explicitly tailored for diabetic patients are imperative. Such guidelines should integrate evidence from robust clinical trials, meta-analyses, and systematic reviews, ensuring standardized, practical, and individualized protocols that are feasible for broad clinical adoption. This targeted approach would substantially enhance procedural efficiency, reduce healthcare-associated costs, and markedly improve patient safety, satisfaction, and clinical outcomes. The development of an evidence-based, standardized bowel preparation protocol specific to diabetic patients would not only enhance procedural safety and diagnostic yield but also improve long-term colorectal cancer prevention. As global diabetes prevalence continues to rise, optimizing colonoscopy effectiveness among diabetic patients becomes increasingly critical for effective colorectal cancer screening and prevention. Therefore, prioritizing focused clinical research and evidence-based guideline development aimed explicitly at addressing diabetic patients’ unique bowel preparation challenges must remain a paramount objective within gastroenterological practice and healthcare policy.

## 6. Conclusions

Diabetic patients consistently exhibit inferior bowel preparation outcomes, which may jeopardize the diagnostic efficacy of colonoscopy and delay colorectal cancer detection. Current clinical guidelines are insufficiently tailored to this high-risk group. Our findings underscore the urgent need for prospective trials, new preparation formulations, and clear, evidence-based protocols that account for the unique physiological and pharmacological challenges of diabetes mellitus. Prioritizing such tailored approaches is essential to improving clinical outcomes, patient safety, and resource utilization in colorectal cancer prevention.

## Figures and Tables

**Table 1:** List of studies/research on the topic of bowel preparation for colonoscopy in diabetics.

Authors/Year of Publication	Type of Study	Study Population Number of Participants/N	Results
Abu-Freha N et al., 2025. [7]	multicenter retrospective study	4876 patients treated with GLP-1Ras 4876 controls without GLP-1RA use	Among the GLP-1RA patients, 10% (*n* = 487) had IBP compared to 197 (4%) in the control group (*p* < 0.001). Higher rate of IBP among diabetic patients treated with GLP-1RA (284/2364 [12%]) than among diabetic patients without GLP-1RA treatment (118/2364 [5%]; *p* < 0.001). Diabetes and GLP-1RA use were both found to be independent risk factors for IBP.
Zhang Y et al., 2024. [8]	systematic review and meta-analysis	six studies (*n* = 1553) on previous abdominal surgery, six studies (*n* = 1494) on constipation, seven studies (*n* = 1505) on diabetes, eight studies (*n* = 2093) on non-compliance with the diet regimen, seven studies (*n* = 1350) on incomplete intake of laxative, and nine studies (*n* =2163) on inadequate exercise during preparation.	History of abdominal surgery, constipation, diabetes, non-compliance with the diet regimen, incomplete intake of laxatives, and inadequate exercise during preparation were independent risk factors for IBP in older patients undergoing colonoscopy.
Adamek HE et al., 2022. [9]	review	many studies	Split dosing of PEG preparations is recommended in diabetes patients with expected motility disorders. Extensive counseling about preparation, intake, and dietary recommendations should be offered.
Zhao M et al., 2022 [10]	prospective cohort study	N = 436	The highest ADR was achieved when the WT of colonoscopy was controlled at 8 min.
Lewandowski K et al., 2021. [11]	review	many studies, without final count of study population	Patients with DM are particularly predisposed to inadequate cleansing for endoscopy due to slowed bowel movements, dietary preparation restrictions, glucose reduction, and the resulting symptoms of hypoglycemia. No comprehensive guidelines for the preparation of endoscopic examinations for patients with DM have been developed.
Agha OQ et al., 2021. [12]	review	many studies	DM is associated with suboptimal bowel preparation for colonoscopy. Several studies attempted to optimize bowel preparation in these patients. However, these studies vary in the strength of their evidence, and most of them did not use split-dosing regimens, which are part of the current ASGE recommendation.
Ruiz RF et al., 2020. [13]	randomized controlled trial	N = 100 participants	Colonoscopy was performed after upper digestive endoscopy at two different times: 3 versus 6 h after 10% mannitol ingestion. The subgroup of patients with diabetes mellitus showed statistically significant higher RGV values in the 3 h group.
Fuccio L et al., 2020. [14]	prospective observational study	N = 1032 participants	Bedridden status, constipation, diabetes mellitus, use of anti-psychotic drugs, and 7 or more days of hospitalization increased the risk of inadequate colon cleansing.
Hochberg I et al., 2019. [15]	review	many studies	To prevent the risk of hypoglycemia, hyperglycemia, and ketoacidosis lactic acidosis, and to improve bowel preparation in people with DM, clear guidelines should be provided regarding diet, medication timing, and glucose monitoring. There is evidence that mid-morning scheduling (after 9:30 a.m.) improves bowel preparation in patients with DM as it facilitates adherence to split dosing of the laxative.
Megna B et al., 2018. [16]	observational study	N = 88 participants	Risk factors, such as older age, history of DM, the timing and split dosing of preparation solution, procedure time (AM or PM), chronic narcotic use, and history of constipation, for inadequate bowel preparation were not associated with the ability to perform CE.
Mahmood S et al., 2018. [17]	meta-analysis	twenty-four studies with a total of 49,868 patients	Age, male sex, inpatient status, DM, hypertension, cirrhosis, narcotic use, constipation, stroke, and tricyclic antidepressant use were associated with inadequate bowel preparation.
Anklesaria AB et al., 2019. [18]	observational Study	N = 1429 patients	Male gender (*p* = 0.002), diabetes mellitus (*p* < 0.0001), liver cirrhosis (*p* = 0.001), coronary artery disease (*p* = 0.003), refractory constipation (*p* < 0.0001), and current smoking (*p* = 0.01) were found to be independently predictive of poor bowel preparation.
Mandolesi D et al., 2017. [19]	review	many studies	The quality of colonoscopy has become a hot topic. The approach to patients with an increased risk of poor bowel preparation quality is still not always supported by high-quality evidence. Trials focused on this subgroup of patients are recommended to provide tailored bowel preparation regimens and guarantee high-quality procedures.
Alvarez-Gonzalez MA et al., 2016. [20]	randomized controlled trial	N = 150 patients with type 2 DM N = 74 conventional bowel preparation protocols (CBPs) versus N = 76 diabetes-specific preparation protocols (DSPs)	Inadequate bowel cleansing was more frequent following CBPs than DSPs (20% vs. 7%, *p* = 0.014).
Park JS et al., 2016. [21]	randomized controlled trial	N = 520 patients	Males, DM, and non-use of visual aids were associated with poor bowel preparation. The addition of an educational video could improve the quality of bowel preparation in comparison with the standard preparation method.
Kim YH et al., 2017. [22]	randomized controlled trial	N = 55 consecutive non-diabetic and N = 50 diabetic patients	Diabetic patients had a worse preparation quality and longer cecal intubation and total procedure time compared to non-diabetic patients. These data suggest that the split-dose PEG preparation regimen is not sufficient for optimal bowel preparation in diabetic patients undergoing colonoscopy.
Rotondano G et al., 2015. [23]	prospective multicenter study	2178 outpatients, 1098 inpatients	In the overall population, independent predictors of inadequate cleansing both at the level of right and left colon were as follows: male gender, diabetes mellitus, chronic constipation, incomplete purge intake, and a runway time >12 h. No differences in the rate of inadequate bowel preparation between hospitalized patients and outpatients were found.
Hayes A et al., 2011. [24]	randomized controlled trial	198 persons with DM	Patients in the diabetic colon preparation group had 70% good colon preparations compared to 54% in the standard group, and this finding was significant (χ = 5.14, *p* = 0.02). The results indicate that diabetic patients receiving 10 ounces of magnesium citrate 2 days prior to their colonoscopies followed by 10 ounces of magnesium citrate and 4 L polyethylene glycol the day prior to the procedure had cleaner colons than those receiving standard preparation of 10 ounces of magnesium citrate and 4 L polyethylene glycol the day prior to procedure. This colon preparation is safe, feasible, well tolerated, and effective.
Ozturk NA et al., 2010. [25]	controlled clinical trial	50 consecutive type 2 diabetic patients and 50 non-diabetic patients	Data suggest that NaP is safe and tolerable in diabetic patients, but the quality of bowel cleansing is worse than in non-diabetic patients. These observations support the concept that the quality of bowel cleansing in those with type 2 diabetes is closely related to the duration and regulation of the disease and the presence of late complications.
Ozturk NA et al., 2009. [26]	clinical trial	45 patients with DM and 48 non-diabetic	These data suggest that optimal bowel cleansing is poorer in diabetics with autonomous neuropathy than in those without autonomous neuropathy and controls. Although optimal bowel cleansing was more prevalent among control patients than in diabetic patients without autonomous neuropathy, the difference was not significant (87.1% vs. 93.8%; *p* > 0.05).

Legends: GLP-1Ras—glucagon-like peptide-1 receptor agonist; IBP—inadequate bowel preparation for colonoscopy (“poor preparation” on Aronchik scale or Boston Bowel Preparation Scale < 5); PEG—polyethylene glycol; ADR—adenoma detection rate; WT—colonoscopy withdrawal time; DM—diabetes mellitus; ASGE—American Society of Gastrointestinal Endoscopy; RGV—residual gastric volume; CE—chromoendoscopy; NaP—sodium phosphate.

**Table 2:** Current guidelines for bowel preparation for colonoscopy.

Guidelines/Organization (Reference)	Date of Publication	Specific Recommendations for Bowel Preparation for Diabetic Patients (YES/NO/Mentioned but Without Clear Guidelines)
European Society of Gastrointestinal Endoscopy (ESGE) Guideline [27]	2019	NO
US Multi-Society Task Force on Colorectal Cancer: American College of Gastroenterology, American Gastroenterological Association, and American Society for Gastrointestinal Endoscopy [28]	2014	mentioned but without clear guidelines
Canadian Association of Gastroenterology [29]	2006	NO
